# Evolution of olfactory circuits in insects

**DOI:** 10.1007/s00359-020-01399-6

**Published:** 2020-01-27

**Authors:** Zhilei Zhao, Carolyn S. McBride

**Affiliations:** 1 Department of Ecology and Evolutionary Biology, Princeton University, Princeton, NJ 08544 USA; 2 Princeton Neuroscience Institute, Princeton University, Princeton, NJ 08544 USA

**Keywords:** Evolution, Olfaction, Neural circuits, Insects, *Drosophila sechellia*

## Abstract

Recent years have seen an explosion of interest in the evolution of neural circuits. Comparison of animals from different families, orders, and phyla reveals fascinating variation in brain morphology, circuit structure, and neural cell types. However, it can be difficult to connect the complex changes that occur across long evolutionary distances to behavior. Luckily, these changes accumulate through processes that should also be observable in recent time, making more tractable comparisons of closely related species relevant and complementary. Here, we review several decades of research on the evolution of insect olfactory circuits across short evolutionary time scales. We describe two well-studied systems, *Drosophila sechellia* flies and *Heliothis* moths, in detailed case studies. We then move through key types of circuit evolution, cataloging examples from other insects and looking for general patterns. The literature is dominated by changes in sensory neuron number and tuning at the periphery—often enhancing neural response to odorants with new ecological or social relevance. However, changes in the way olfactory information is processed by central circuits is clearly important in a few cases, and we suspect the development of genetic tools in non-model species will reveal a broad role for central circuit evolution. Moving forward, such tools should also be used to rigorously test causal links between brain evolution and behavior.

## Introduction

An animal’s fitness depends heavily on how it behaves in response to environmental stimuli. Behavior is, therefore, subject to strong selection and often evolves during colonization of a new ecological or social niche. Despite the ubiquity of such changes, we know very little about their proximate neural mechanisms. On the one hand, evolutionary biologists have been studying how and why behavior evolves at the organismal level for over a century (Darwin 1859; Alcock 2013). On the other hand, neurobiologists have worked over the same period to develop a mechanistic understanding of how behaviors are regulated at the level of neural circuits in model systems (Kandel 2013; Luo 2015). However, work at the intersection of these two fields, evolution and neurobiology (evo-neuro), is only just beginning to accelerate (e.g*.*, de Bono and Bargmann 1998; Lim et al. 2004; Newcomb and Katz 2009; Prieto-Godino et al. 2017; Bendesky et al. 2017; Seeholzer et al. 2018; Ding et al. 2019; Hong et al. 2019). How do neural circuits themselves evolve to drive adaptive behavior in a new environment?

Early progress in a field is often made through detailed study of a simple system. The study of olfactory behavior in insects partly fills this role for evolutionary neurobiology. The ways in which olfactory circuits change through time and differ between closely related insect species has received more attention than almost any other area of inquiry. There are several reasons for this. First, insects have simpler brains than vertebrates and are usually easier to rear and manipulate in the laboratory. Second, sensory input at the periphery of the insect olfactory system can be monitored very easily via electrophysiology. Third, insects rely heavily on their sense of smell for both innate and learned behavior (Menini 2009; Hansson 2013). Odors often serve as primary cues for attracting mates, avoiding parasitoids, foraging, and egg-laying. They are also the main substrate for insect learning and memory (Davis 2005; Giurfa and Sandoz 2012). Olfactory behaviors are, therefore, among the first behaviors to evolve as insects adapt to new environments. Add to this the huge diversity of insect species, and you have a near infinite supply of clear, tractable examples for study (Nei et al. 2008; Stensmyr 2009; Ramdya and Benton 2010; Hansson and Stensmyr 2011; Andersson et al. 2015). Finally, the neural circuits that underlie olfaction are relatively well characterized in model insect species (Menini 2009; Hansson 2013). This means that researchers can quickly dive into questions of circuit evolution without first having to do decades worth of careful background study.

Here, we review work on the evolution of olfactory circuits in insects. We ask whether it is possible to identify general patterns in the types of circuit changes that tend to be selected during evolution in natural populations, and the position of those changes within a circuit. We focus narrowly on evolution over short evolutionary time scales—between closely related populations or species. In this setting, homologous neurons are easier to identify and observed changes are more likely to be causal. However, there is also an interesting literature on differences among more distantly related taxa (Hansson and Stensmyr 2011, Strausfeld and Hildebrand 1999, Farris 2011), which is not covered here. We begin with a brief review of olfactory circuit organization followed by detailed case studies of evolution in *Drosophila sechellia* flies and *Heliothis* moths. We then move more systemically through the different ways in which evolution may tinker with olfactory circuits, bringing in examples from other insects, including other *Drosophila* and moth species, mosquitoes, social bees, and wasps. Although most of the examples we describe are linked to behavior in some way (e.g., via the ecological relevance of key ligands), we caution that almost all are still correlational. Only very recently have we seen a clear demonstration of causality for one of many changes in the *Drosophila sechellia* system (Auer et al. 2019).

## Organization of insect olfactory circuits

**Fig. 1:**
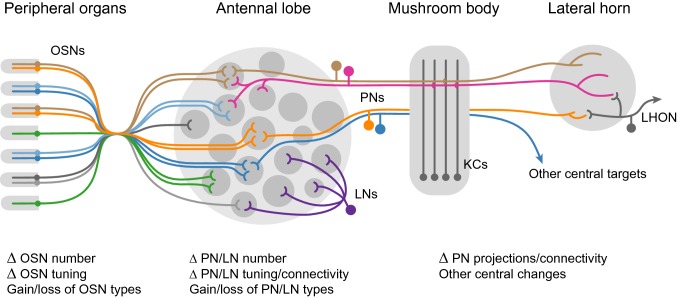
Basic organization of insect olfactory circuits. Left, olfactory sensory neurons (OSNs) are housed in sensilla scattered across antennae and other peripheral organs. Middle, OSNs send axons to the antennal lobe. All OSNs that express the same ligand-specific receptor converge onto a single glomerulus where they synapse with projection neurons (PNs) and local interneurons (LNs). Most PNs innervate only one glomerulus (brown, orange, blue), but some are multiglomerular (pink). LNs tend to innervate many, if not all, glomeruli (purple). Right, PNs send axons to higher brain centers. Many synapse on Kenyon cells (KCs) in the mushroom body calyx before passing on to the lateral horn (brown, pink). Others project directly to the lateral horn (orange) or other brain areas (blue). Diverse lateral horn neurons, including lateral horn output neurons (LHONs) may integrate information coming from multiple PN populations to drive innate behaviors. Below the diagram, we list a few of the many types of changes that could occur at each circuit level during evolution

Olfaction in insects begins when a volatile compound diffuses into porous hair-like structures called sensilla scattered across the antennae and other olfactory organs (Menini 2009; Hansson 2013). Each sensillum houses one or more olfactory sensory neurons or OSNs (Fig. 1). If the compound is recognized by an olfactory receptor complex in the membrane of one of these OSNs, binding may trigger the neuron to fire, sending a signal to the brain. With exceptions, each OSN expresses only one tuning receptor in addition to one or more co-receptors. It is the tuning receptor that largely determines the set of odorants to which a neuron will be sensitive. However, OSN dendrites are bathed in an extracellular lymph that contains secreted accessory proteins, such as odorant-binding proteins (OBPs). The role of these proteins, and OBPs in particular, is still unclear (Leal 2013; Brito et al. 2016; Larter et al. 2016), but they may regulate OSN responses by affecting the rate at which odorants diffuse into, or are cleared from, sensilla. Importantly, there are different classes of sensilla and each class houses a stereotyped combination of OSNs (Fig. 1). For example, each sensillum belonging to a given class might house one OSN expressing receptor X and another expressing receptor Y.

The gross organization of higher olfactory circuits is well conserved across neopteran insects (Strausfeld and Hildebrand 1999). OSNs carry olfactory information from the periphery to an area of the brain called the antennal lobe (Fig. 1). Within this region, all OSNs that express the same receptor(s) converge on a single structural unit called a glomerulus (Vosshall and Stocker 2007). Odors activate specific subsets of receptors, and, therefore, specific subsets of glomeruli, producing a combinatorial glomerular code that is thought to underlie olfactory discrimination (Galizia et al. 1999; Wang et al. 2003). Within glomeruli, OSNs synapse onto second-order neurons such as local interneurons and projection neurons. Most excitatory projection neurons (PNs) are uniglomerular; they receive information from a single glomerulus and relay it to higher centers. Each glomerulus thus serves as a distinct information channel, albeit not completely independent from other glomeruli due to the complex network of local interneurons that implement transformations such as gain control (Wilson 2013). However, multiglomerular PNs are also common (Homberg et al. 1988; Stocker et al. 1990). This basic circuit architecture is surprisingly similar to that of vertebrate olfactory systems, representing a classic case of convergent evolution (Strausfeld and Hildebrand 1999; Eisthen 2002).

From the antennal lobe, PNs carry olfactory information to higher centers in the brain, including, but not limited to, the mushroom body and lateral horn (Fig. 1) (Tanaka et al. 2012). PN innervation of mushroom body neurons is sparse and mostly random, consistent with the critical role this area plays in olfactory learning and memory (Murthy et al. 2008; Caron et al. 2013). In contrast, PN arborization in the lateral horn is highly stereotyped, consistent with a primary role in innate olfactory responses (Jefferis et al. 2007). Recent work has made great strides in uncovering the structure and function of circuits in both these areas (Cohn et al. 2015; Frechter et al. 2019; Jeanne et al. 2018), but much remains unknown. It is also important to remember that much of what we know about the structure and function of olfactory circuits in insects comes from careful study of just a handful of species. For example, seminal early work was conducted in the moth *Manduca sexta* (e.g., Homberg et al. 1988), locusts (e.g., Laurent and Davidowitz 1994), and bees (e.g*.*, Galizia et al. 1999), while more recent studies have often taken advantage of the genetic tools available in *Drosophila*. Many principles apply broadly, but there are also exceptions (e.g*.*, the distinct antennal lobe circuitry of many orthopterans; Ignell et al. 2001).

Given this basic architecture, how might we expect olfactory circuits to evolve? In Fig. 1, we provide a non-exhaustive list of different types of circuit changes that may be selected during behavioral evolution. We will systematically review the evidence for each type of change (or lack thereof) after presenting two case studies.

## Case study: evolution of host odor preference in *Drosophila sechellia*

**Fig. 2:**
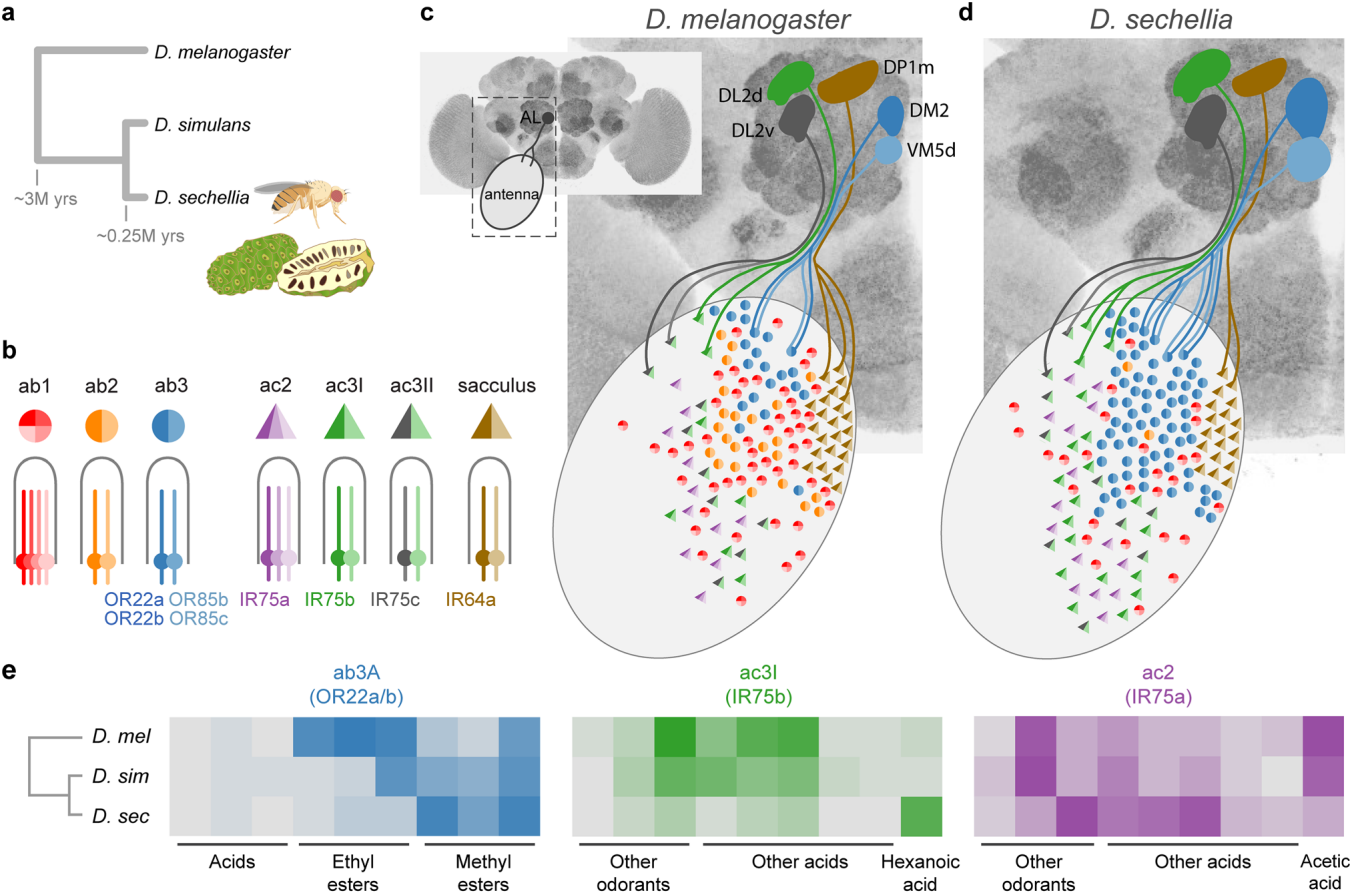
Evolution of peripheral olfactory circuits in *Drosophila sechellia*. **a** Phylogenetic relationships among three closely related *Drosophila*, showing *D. sechellia* and its preferred noni fruit. **b** Types of sensilla and associated OSNs that are discussed in the text and depicted in panels c, d. Receptor names shown for only a subset of cells. Note that *Or22b* is co-expressed with *Or22a* in the ab3A neuron of *D. melanogaster* but is a pseudogene in *D. sechellia*. Conversely, *Or85c* is co-expressed with *Or85b* in the ab3B neuron of *D. sechellia*, but possibly not in *D. melanogaster*. Receptor-OSN pairing is otherwise conserved. **c**, **d**, Schematics showing divergence in sensilla/OSN number and glomerulus size between *D. melanogaster* (**c**) and *D. sechellia* (**d**). Populations of sensilla that have expanded include ab3 (blue circles) and ac3I (green triangles). Those that have contracted or been almost completely lost include ab1 (red circles), ab2 (orange circles), and sacculus neurons (brown triangles). Axonal projections leading to glomeruli in the AL are only shown for a subset of key OSNs. The position and abundance of sensilla are adapted from Dekker et al. (2006), Shanbhag et al. (1999), and Prieto-Godino et al. (2017). **e** Heatmaps showing divergence in sensillum/OSN tuning between *D. melanogaster*, *D. simulans*, and *D. sechellia*. Names of relevant receptor proteins are shown in parentheses below sensillum/OSN name. In each case, either sensitivity to key components of noni fruit odor has increased (e.g., ab3A and ac3I) or sensitivity to odorants that were ancestrally important but emitted by ripe noni in only trace amounts have decreased (e.g., ac2). Data are adapted from Prieto-Godino et al. (2017) and Auer et al. (2019)

*Drosophila sechellia* diverged from its famous relative *Drosophila melanogaster* approximately three million years ago, and from its more closely related sister species *Drosophila simulans* approximately 250,000 years ago (Fig. 2a) (Garrigan et al. 2012). While *D. melanogaster* and *D. simulans* are globally distributed generalists, *D. sechellia* is endemic to the Seychelles archipelago and has evolved to specialize in eating and laying eggs on the toxic fruit of *Morinda citrifolia*, commonly known as noni (Fig. 2a) (Tsacas and Bächli 1981; R’Kha et al. 1991). Specialization in *D. sechellia* is associated with a suite of novel physiological and behavioral traits, including robust attraction to noni odor. *D. sechellia* is strongly attracted to noni odor, while both *D. melanogaster* and *D. simulans* are neutral or only slightly attracted (Auer et al. 2019). How have olfactory circuits evolved to mediate this shift?

### Increased ‘gain’ on peripheral neurons that mediate attraction to noni fruit odorants

The first glimpse of olfactory circuit evolution in *D. sechellia* came from a study by Stensmyr et al. (2003). The authors used electrophysiology to characterize the response profiles of OSNs housed in large basiconic sensilla on the antennae of *D. sechellia*, *D. melanogaster*, *D. simulans*, and six other related species. There are three types of large antennal basiconic sensilla in *D. melanogaster*: ab1, ab2, and ab3 (Fig. 2b). Each houses two or more OSNs that express receptors in the odorant receptor (OR) family and can be recognized by characteristic ligand binding profiles. The three types are approximately equally abundant in *D. melanogaster* and this arrangement was conserved in *D. simulans*. However, *D. sechellia* antennae had half as many ab1, no detectable ab2, and over twice as many ab3 sensilla (Stensmyr et al. 2003) (Fig. 2c, d). This striking change was subsequently confirmed by other studies (Dekker et al. 2006; Auer et al. 2019; Keesey et al. 2019; note ab2 sensilla are sometimes present in *D. sechellia* in very small numbers). Although the altered proportions have not yet been shown to directly affect behavior, it is telling that both neurons housed within the expanded ab3 sensillum population respond to noni odorants that attract *D. sechellia*, but are neutral or repellant to *D. melanogaster* and *D. simulans* (methyl hexanoate and *2*-heptanone; Dekker et al. 2006; Ibba et al. 2010). Moreover, knock-out of either of the corresponding receptors in *D. sechellia* (*Or22a* and *Or85b/c*) completely eliminates long-range attraction to noni juice (Auer et al. 2019).

Interestingly, ab3 sensilla have also changed in tuning. Its two resident neurons are referred to as ab3A and ab3B, or simply A and B. The A neuron is *more* sensitive to methyl esters and *less* sensitive to ethyl esters in both *D. sechellia* and *D. simulans* relative to *D. melanogaster* (Stensmyr et al. 2003; Dekker et al. 2006; Auer et al. 2019) (Fig. 2e). The shift is clearly driven by coding evolution of the resident receptor OR22a and mirrors the composition of noni odor, which has an unusually high ratio of methyl to ethyl esters (Auer et al. 2019). However, the fact that the change is shared between *D. sechellia* and its generalist sibling *D. simulans* suggests it predates noni specialization and previously cast doubt on its relevance for behavior. A recent study finally clarified the situation*. D. sechellia* flies expressing the *D. melanogaster* copy of *Or22a* in the ab3A neuron (instead of the native *D. sechellia* copy) had significantly reduced long-range attraction to noni (Auer et al. 2019). This is the most direct demonstration of a link between any specific case of olfactory circuit evolution and behavior. It was enabled by the development of transgenic tools in *D. sechellia* as well as the relatively simple genetic basis of the tuning shift (OR22a coding changes). It remains unclear why the change may have evolved before the split between *D. sechellia* and *D. simulans*. Unlike neuron A, the B neuron within ab3 sensilla appears to have conserved tuning. It responds to the minor noni volatile *2*-heptanone (Stensmyr et al. 2003; Ibba et al. 2010; Auer et al. 2019).

A second, surprisingly parallel set of changes in OSN number and tuning was recently discovered among coeloconic sensilla. Coeloconic sensilla are morphologically and functionally distinct from basiconic sensilla. They typically house 2–3 neurons that express receptors in the ionotropic receptor (IR) family and are thus tuned to amines and acids (Silbering et al. 2011). A recent study found that *D. sechellia* has twice as many ac3I sensilla as *D. melanogaster* and *D. simulans* (Fig. 2c, d) and that one of the two neurons housed therein shows a striking increase in sensitivity to hexanoic acid (Fig. 2e, middle) (Prieto-Godino et al. 2017). Hexanoic acid is one of the most abundant components of noni odor (Farine et al. 1996; Auer et al. 2019), and, just like the two ligands for ab3 sensilla, it attracts *D. sechellia* while being neutral or repellant to *D. melanogaster* and *D. simulans* (Dekker et al. 2006; Prieto-Godino et al. 2017). The receptor responsible for this shift in tuning is IR75b (Prieto-Godino et al. 2017). There is not yet any direct evidence that changes in ac3I number or tuning affect behavior, but complete knock-out of *Ir75b* does. It reduces short-range attraction of *D. sechellia* to noni juice (Auer et al. 2019).

### Decreased ‘gain’ on peripheral neurons that drove ancestors away from noni

While evolution has increased the gain on sensory neurons that mediate attraction to noni, it has decreased the gain on two other OSN populations. Interestingly, one of these may have driven ancestors away from noni. Ir64a-expressing OSNs are housed in sensilla on a part of the antenna called the sacculus (Fig. 2b) and mediate aversion to acids, including noni acids, in *D. melanogaster* (Ai et al. 2010). They have become somewhat less numerous in *D. sechellia* (Fig. 2c, d) (Prieto-Godino et al. 2017). Another population of neurons with decreased gain in *D. sechellia* may have drawn ancestors towards substrates this species no longer uses. Acetic acid is a major product of fermentation and thus characterizes the rotting substrates favored by *D. sechellia*’s close relatives. Ripe fruit, in contrast, contain less acetic acid, with only trace amounts present in ripe noni (Auer et al. 2019). It is, therefore, suggestive that Ir75a-expressing neurons in ac2 sensilla of *D. sechellia* (Fig. 2b) show reduced sensitivity to this key compound (Fig. 2e, right) (Prieto-Godino et al. 2016).

### Central changes in the noni odor circuit

Soon after researchers first documented striking changes in the number of specific types of sensory neurons on *D. sechellia* antennae, it became clear they were correlated with changes in the size of corresponding glomeruli in the antennal lobe. The three glomeruli targeted by expanded neural populations have increased in volume (DM2, VM5d, DL2d; Fig. 2c, d), while one glomerulus targeted by the diminished population of Ir64a-expressing, acid-sensitive OSNs has shrunk slightly (DP1m; Fig. 2c, d) (Dekker et al. 2006; Ibba et al. 2010; Prieto-Godino et al. 2017). These changes are most likely a direct by-product of OSN numbers and, therefore, peripheral in nature; OSNs are much more numerous than the second-order neurons with which they are connected and, therefore, largely determine glomerulus size (Grabe et al. 2016). Moreover, we know the number of second-order projection neurons has *not* changed for at least two of the key glomeruli (DM2 and DL2d) (Prieto-Godino et al. 2017; Auer et al. 2019). Nevertheless, it is difficult to rule out the potential contribution of synapse density or local neuron number. We also note that one glomerulus appears to have grown in *D. sechellia* without a corresponding change in the number of incoming OSNs (DL2v, receiving input from Ir75c-expressing neurons in ac3II; Fig. 2b–d) (Prieto-Godino et al. 2017).

Auer et al. (2019) recently characterized another change that is indisputably central in origin. They found a novel branch on the axons of DM2 projection neurons within the lateral horn. These neurons carry information from the expanded population of methyl ester-sensitive ab3A OSNs, raising the intriguing possibility that the way in which noni-evoked signals are integrated and ascribed innate meaning in the brain of *D. sechellia* has also evolved. It will be exciting to see future work explore this possibility.

In summary, evolution has reshaped the periphery of *D. sechellia*’s olfactory system by tinkering with OSN number and sensitivity in ways that appear to increase the gain on neurons that mediate attraction to noni fruit and, perhaps to a lesser degree, decrease the gain on neurons that helped steer ancestors away from noni and toward other substrates. Future work will hopefully find creative ways to test the hypothesis that central circuit changes also contribute.

## Case study: Evolution of pheromone preference in *Heliothis* moths

Moths have long been a model system for insect olfaction (Haupt et al. 2011). Females release species-specific volatile pheromones that males use to locate them (Cardé and Cardé 2016). Female moth pheromones usually include a mix of several compounds. For some species, a single component from the mixture can be behaviorally attractive (e.g*.*, *Bombyx mori*; Butenandt et al. 1959), while other species require a blend with appropriate ratios (e.g*.*, *Manduca sexta*; Tumlinson et al. 1989). OSNs that are sensitive to pheromones are housed in the long trichodea sensilla on male antennae and project to a special region in the antennal lobe called the macroglomerular complex (MGC) (Matsumoto and Hildebrand 1981; Kankazi and Shibuya 1986). The MGC is male-specific and segregated from other glomeruli.

**Fig. 3:**
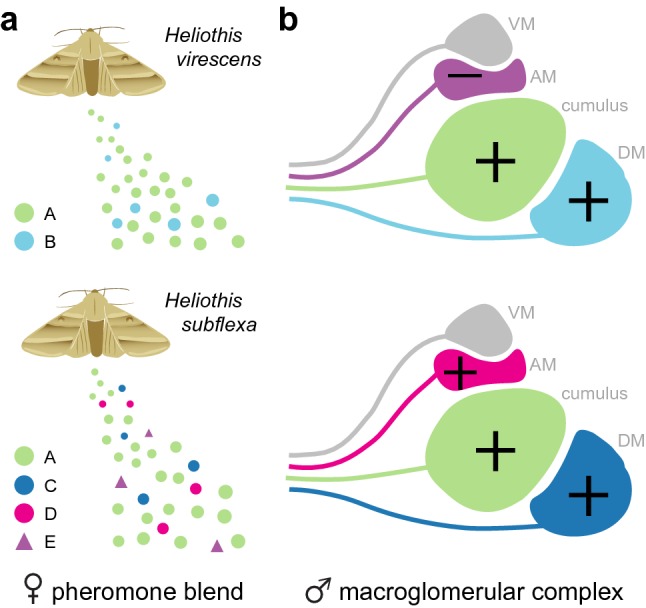
Evidence for peripheral and central circuit evolution in *Heliothis* moths. **a** Female pheromone blends of *H. virescens* and *H. subflexa* share major component A but differ in secondary components B–E. Circles or triangles represent compounds that promote or inhibit attraction, respectively, in conspecific and/or heterospecific females. A, (Z)-11-hexadecenal; B, (Z)-9-tetradecenal; C, (Z)-9-hexadecenal; D, (Z)-11-hexadecenol; E, (Z)-11-hexadecenyl acetate. **b** Schematic of the macroglomerular complex of each species. Glomerulus color indicates tuning for ligands in panel a. Symbols indicate valence—i.e.*,* whether signaling in each glomerulus has a positive or negative effect on behavior. Note that glomerulus AM has negative valence in *H. virescens* but positive valence in *H. subflexa*, indicating there must be a change in downstream circuits. Data are adapted from Vickers and Christensen (2003) and Baker et al. (2004)

Like many sexual signals, female pheromone blends and male preferences evolve rapidly. For example, *Heliothis virescens* and *Heliothis subflex*a are closely related noctuid moths with pheromone blends that share the same major compound but a distinct set of secondary compounds (Fig. 3a) (Roelofs et al. 1974; Teal et al. 1981; Vickers 2002). For readability, we will use A to refer to the major compound and B, C, D, and E to refer to the various secondary compounds. The legend of Fig. 3 lists their full names. *H. virescens* females release both A and B (Fig. 3a, top), and males require both for attraction. *H. subflexa* females release A, C, D, and E (Fig. 3a, bottom), but males only require A, C, and D for attraction. Compound E repels heterospecific *H. virescens* males, and thus may have been selected to prevent maladaptive hybridization. Careful study of this system has revealed clear evidence for evolution of both peripheral and central pheromone circuits.

### Combination of peripheral and central changes

The morphology of the MGC in *H. virescens* and *H. subflexa* is essentially indistinguishable (Fig. 3b). There are four major MGC glomeruli: a large glomerulus called cumulus and three smaller surrounding glomeruli called DM, AM, and VM (Hansson et al. 1995; Vickers et al. 1998; Berg et al. 1998; Vickers and Christensen 2003). Electrophysiological recordings in OSNs and PNs followed by dye-filling revealed the tuning of each glomerulus. The large cumulus is sensitive to the major pheromone component A in both species (Fig. 3b), indicating that both its tuning and valence are conserved. Valence refers to the behavioral effect of signaling in the given neuropil and is positive in this case, since compound A drives attraction in both species. Interestingly, the tuning of DM has changed. It responds to B in *H. virescens*, but to the related compound C in *H. subflexa* (Fig. 3b). This reflects a change in OSN tuning at the periphery; homologous OSNs are sensitive to B in one species but to C in the other (Baker et al. 2004). Importantly, however, B and C both have a positive effect on behavior in their respective species, so valence is again conserved. The change in tuning has been mapped to an area of the genome containing several pheromone receptors (Gould et al. 2014).

From the perspective of central circuit evolution, the most interesting glomerulus is AM. We again see a tuning shift that can likely be traced to changes at the periphery. Neurons in AM respond to E in *H. virescens* and the related D compound in *H. subflexa* (Fig. 3b). More importantly, however, this time there has also been a shift in valence. Compound E inhibits upwind flight in *H. virescens* while compound D stimulates it in *H. subflexa* (Fig. 3b). The behavioral effect of signaling in a given glomerulus should be a function of downstream circuitry. The change in AM valence thus strongly suggests that the downstream circuits have evolved. Antennal transplant experiments provide corroborating evidence (Lee et al. 2016). In these remarkable manipulations, the pupal antennal disc that holds OSN progenitors and will eventually develop into the antenna is surgically swapped between individuals. Failure rate is high, but when it works, the result is an insect with peripheral neurons from one moth and central circuits from another. Lee et al. (2016) transplanted antennal discs from *H. virescens* donors to both conspecific and heterospecific recipients. Interestingly, inputs to only two glomeruli were required to initiate flight in conspecific recipients, whose central circuits came from *H. virescens*. In contrast, inputs to three glomeruli were required in heterospecific recipients, whose central circuits were derived from *H. subflexa* (Lee et al. 2016). Further work is needed to identify the nature of the underlying central changes.

## Evolution of peripheral olfactory circuits

*Drosophila sechellia* and *Heliothis* moths provide two of the most thoroughly characterized examples of olfactory circuit evolution in insects. They show that many diverse changes can accumulate in a single taxon over relatively short evolutionary time scales. We now turn to a more systematic review of the types of peripheral changes that have been observed across insects more broadly. These fall into three categories: changes in OSN tuning (with potentially diverse underlying mechanisms), changes in OSN number, and the complete gain or loss of OSN types (Fig. 1).

### Changes in OSN tuning via receptor protein evolution

One of the most common ways in which peripheral olfactory systems evolve is through changes in sensory neuron tuning. The literature provides numerous examples, most of which involve an increase in sensitivity to compounds with new social or ecological relevance. This was true for the observed tuning shifts in *Heliothis* moths, described above. Indeed, the rapid evolution of moth pheromones, and the often one-to-one link between female pheromone components and the male OSN populations that detect them, make tuning evolution a particularly ubiquitous feature of moth pheromone detection systems. Beyond *Heliothis*, examples include species in the genera *Ctenopseustis* (Hansson et al. 1989), *Agrotis* (Hansson et al. 1990), *Trichoplusia* (Domingue et al. 2009), *Ostrinia* (Leary et al. 2012), and *Helicoverpa* (Yang et al. 2017).

Tuning evolution is also a common feature of host detection systems in the genus *Drosophila*, and one neuron in particular is emerging as a hotspot for such changes. The ab3A neuron that shifted in *D. sechellia* also shows increased sensitivity to host-associated compounds in wild, ancestral populations of *D. melanogaster* that specialize on fallen marula fruit (Mansourian et al. 2018; see also Shaw et al. 2019) and in the distantly related *D. suzukii* that lays eggs on ripe fruit (Keesey et al. 2015). Moreover, ab3A tuning was uniquely variable in a recent tour-de-force survey of odor responses in 10 OSN types across 20 *Drosophila* species (Keesey et al. 2019). Most neurons showed conserved tuning across the clade, while ab3A flipped back and forth in whether it was most sensitive to methyl esters, ethyl esters, isobutyl acetate, or β-cyclocitral. The authors suggest the changes may reflect repeated adaptation to rotting substrates (characterized by the methyl and ethyl esters) from ancestors that preferred ripe fruit. The underlying assumption is that while tuning is labile, the important role ab3A plays as a positive input to a host attraction circuit (Auer et al. 2019) is conserved across the genus.

*Aedes aegypti* mosquitoes also show trends for enhanced neural response to volatiles found in biologically relevant blends. This species, which transmits dengue, zika, and yellow fever, has recently evolved to specialize in biting humans and shows a robust preference for human odor over animal odor (McBride 2016). Preference for humans is associated with an increase in the sensitivity of odorant receptor *AaegOr4* to the abundant human volatile sulcatone (McBride et al. 2014). While a direct connection between receptor evolution and behavior has not yet been established, the two show a strong genetic correlation in F2 hybrids between derived human-preferring and ancestral animal-preferring populations. *AaegOr4* expression was also positively correlated with preference in hybrids (McBride et al. 2014).

The selectivity and sensitivity of insect OSNs are primarily determined by the receptor they express (Hallem et al. 2004), and changes in OSN tuning can thus usually be traced to changes in receptor coding sequences. This is true for many, if not all, of the examples described above. However, other types of changes can also alter tuning, as illustrated in the next section.

### Changes in OSN tuning via receptor-neuron pairing

There are hints in the literature of cases where qualitative changes in receptor expression, or receptor-neuron pairing, also contribute to novel behavior. In *Drosophila*, unlike mice, OSN identity and development is independent of receptor expression. OSNs find targets in the antennal lobe *before* receptors are expressed (Dobritsa et al. 2003; Barish and Volkan 2015). OSNs can, therefore, be classified into discrete types based on their glomerular targets and other intrinsic properties, without reference to receptors. And OSN tuning may thus evolve not only by a change in the *coding sequence* of the expressed receptor, but also by a change in the *identity* of the expressed receptor. One suggestive example again comes from *Drosophila*, where expression of the receptor OR35a in one of two neurons within ac3 sensilla may be limited to the *melanogaster* subgroup (Nemeth et al. 2018). While it is not yet known whether and how this change affects behavior, a second potential example, from the moth *Ostrinia nubilalis*, has clear behavioral effects.

*O. nubilalis*, commonly known as the European corn borer, includes two reproductively isolated races (Klun et al. 1973; Kochansky et al. 1975; Malausa et al. 2005). Females of both races produce a pheromone blend containing the E and Z isomers of 11-tetradecenyl acetate. However, the ratio is 99:1 for E-strain females and 3:97 for Z-strain females (Klun et al. 1973; Kochansky et al. 1975). Males show strong attraction only to the ratio characteristic of their own race. The circuit basis of this shift in male behavior is not fully understood, but several observations point to a change in pairing between pheromone receptors and preexisting OSN types.

Electrophysiological recordings from antennae of males from both races identified a single type of pheromone-sensitive sensillum housing three OSNs (Koutroumpa 2014). The OSNs can be differentiated based on spike amplitude. In E-strain males, the OSN with the largest amplitude responded to the E isomer, while a second neuron with smaller amplitude responded to the Z isomer. The exact opposite was true in Z-strain males—the large amplitude OSN was sensitive to Z and the small amplitude OSN was sensitive to E. In *Drosophila*, spike amplitude depends on intrinsic properties of an OSN rather than the receptor (Hallem et al. 2004). This suggests a change in pairing between receptor and other neuronal properties. Another clue, which was actually the first to be discovered, comes from studies of OSN targets in the macroglomerular complex (MGC) of the antennal lobe (Kárpáti et al. 2008). In corn borers, the MGC contains a larger medial glomerulus and a smaller lateral glomerulus. In E-strain males, the larger glomerulus is sensitive to the E isomer and the smaller glomerulus is sensitive to the Z isomer. In Z-strain males the pattern is reversed. Glomerulus size must not be a function of OSN number in this case, since E- and Z-sensitive OSNs are housed in the same sensillum in a 1-to-1 manner. Instead, the position and size of the glomeruli likely reflect other developmental properties of OSN axons and the second-order neurons they target. Indeed, a potential difference between the two neurons in axon diameter could explain both the variation in spike amplitude and glomerulus size. Again, we see a mismatch between neural tuning—likely a function of receptor identity—and OSN properties/type. It will be exciting to discover how this is regulated at the genetic level and directly test the effect on behavior.

### Changes in OSN tuning via accessory protein evolution

OBPs are accessory proteins secreted into the extracellular lymph that bathes OSN dendrites within sensilla. Their sequence and expression might thus be expected to affect OSN responses, and some clearly play a role in olfaction (Brito et al. 2016). They also often stand out as being differentially expressed in closely related species (Kopp et al. 2008; Dworkin and Jones 2009; Shiao et al. 2015). Nevertheless, the behavioral effects of such changes are far from clear. One functional study in *D. melanogaster* found robust OSN responses even after the sole OBP from the corresponding sensillum had been knocked-out (Larter et al. 2016). Another study identified a role for an OBP in humidity sensing (Sun et al. 2018). It, therefore, remains to be seen how OBPs or other accessory proteins might contribute to the evolution of olfactory behavior (but see Matsuo et al. 2007 for a clear example of an OBP driving the evolution of taste responses).

### Changes in OSN number

Another way in which evolution may tinker with neural circuits is to adjust the number of neurons in preexisting neural populations. In *D. sechellia*, for example, we saw a striking expansion of three OSN populations that mediate attraction to noni volatiles (Fig. 2c, d). Increasing (or decreasing) the number of OSNs that respond to a given compound in this way could serve to enhance (or diminish) detection sensitivity and reliability (Meisami 1989). Such changes are less common in the literature than changes in OSN tuning, but they are also much harder to detect, requiring extensive electrophysiological recording or challenging RNA in situ/antibody staining protocols.

*Drosophila erecta*, another close relative of *D. melanogaster*, is endemic to gallery forests in west-central Africa and specializes on the fruit of several *Pandanus* species (Rio et al. 1983). An electrophysiological survey of summed antennal signals showed that *D. erecta* antennae respond more strongly to the *Pandanus* volatile 3-methyl-2-butenyl acetate (3M2BA) than do the antennae of their closest relatives, and that the enhanced antennal signal is likely driven by an increase in the number, but not sensitivity, of ab3A neurons (Linz et al. 2013). More work is needed to confirm a link between circuit evolution and behavior, but this is the same OSN type that serves as a hotspot for the evolution of novel host preferences across the genus (see above).

Interestingly, in both *D. sechellia* and *D. erecta*, changes in OSN number reflect changes in sensillum number rather than changes in the number of OSNs housed within individual sensilla. There are likely developmental and/or functional constraints that make the former more labile than the latter. For example, tweaking the expression level of a transcription factor involved in lineage decisions could easily shift fate from one sensillum type to another during development (Barish and Volkan 2015). Consistent with this idea, *D. sechellia* appears to have gained ab3 sensilla at the expense of ab2 sensilla (Fig. 2c, d). Moreover, a broad electrophysiological survey of 20 *Drosophila* species showed shifting ratios of all three types of large basiconic sensilla across the genus (Keesey et al. 2019). Species in the *melanogaster* subgroup tended to have the most ab3 (with *D. sechellia* and *D. erecta* representing extreme examples), spotted-wing species tended to have the most ab2, while still others were biased towards ab1.

Finally, like the dengue vector *Aedes aegypti*, the African malaria mosquito *Anopheles coluzzii* has also evolved to specialize in biting humans and shows robust preference for human odor (Takken and Verhulst 2013; McBride 2016). A comparison of antennal gene expression between this species and a close, animal-biting relative suggests that receptors sensitive to human odorants are more likely to show increased expression than those sensitive to compounds not found in human odor (Rinker et al. 2013). This could be a sign that the number of human-sensitive neurons has increased, or that receptor expression has increased *within* neurons. Clarifying this ambiguity, both here and in other systems showing species or population level divergence in organ-level receptor expression (e.g*.*, Kopp et al. 2008; Dworkin and Jones 2009; McBride et al 2014; Shiao et al. 2015), will be important moving forward.

### Gain/loss of OSN types

One type of peripheral change that seems to be quite rare across short time scales is the evolution of new sensory neuron cell types. Distantly related insects often vary dramatically in the number of distinct classes of sensory neurons or sensilla (Hansson 2013). The gain of new cell types must, therefore, be an important evolutionary force across medium to long time scales. Its absence from the systems we review here suggests the process is too rare or gradual to be reliably observed across short time scales. After all, cell type birth requires the evolution of new developmental patterning mechanisms, which can involve multiple coordinated genetic changes that take longer to accumulate (Perry et al. 2016; Arendt et al. 2019).

The two subtypes of ac3 sensilla in *D. melanogaster* may represent an intermediate step in this kind of gradual process. ac3I and ac3II sensilla share a conserved OR35a-expressing neuron, but differ in the second neuron—which expresses either IR75b (ac3I) or IR75c (ac3II) (Fig. 2b) (Prieto-Godino et al. 2017). Interestingly, these two IRs arose via tandem gene duplication ~ 50 million years ago and their resident neurons project to different compartments of the same glomerulus (Prieto-Godino et al. 2017). ab3 sensilla in *D. suzukii* also comprise two putative subtypes (Keesey et al. 2019). The situation evokes a process by which OSN and sensilla types are gained through the gradual acquisition of new receptors, patterning mechanisms and antennal lobe targets for a subset of previously uniform progenitor cells.

Alternatively, one recent study demonstrated how insects may gain OSN types in a more abrupt way—by suppressing programmed cell death. During olfactory development in *Drosophila*, every sensillum is derived from a single precursor cell that divides several times to generate eight terminal cells, four of which have the potential to become OSNs (Barish and Volkan 2015). In all but one sensillum type, however, one or more of these four cells undergoes programmed cell death, resulting in just one-to-three functional OSNs. In a fascinating recent study, Prieto-Godino and colleagues showed that inhibition of programmed cell death in the developing antenna can rescue what are presumed to be at least some of these cells, resulting in new ‘undead’ neurons that express receptors, show odor-evoked activity, and integrate into olfactory circuits in novel ways (Prieto-Godino et al. 2019). This was especially clear in at1 sensilla, which normally house just one narrowly tuned OSN with large spike amplitude. In some of the manipulated flies, at1 gained a second OSN that responded to diverse odorants and had smaller spike amplitude. Whether or not evolution has taken advantage of this hidden potential remains to be seen. However, the authors showed that this same at1 sensillum does indeed house a second neuron with smaller spike amplitude in multiple species scattered across the *Drosophila* phylogeny.

OSN types can presumably also be lost by mechanisms involving programmed cell death or by conversion of one sensillum type to another. For example, our case study illustrated how *D. sechellia* has nearly lost one class of sensillum (ab2) and its two resident OSN types within just the past few hundred thousand years (Fig. 2c, d). Small numbers of ab2 are still observed in some individuals and strains (see Keesey et al. 2019), but it is easy to imagine how loss could eventually become complete.

### Increased investment in olfaction in social insects

A gross comparison of olfactory systems across insects reveals a more general axis of variation. Some species invest more in olfaction than others. Eusocial insects are a prime example of a group that has increased investment in olfactory communication across long evolutionary time scales. Ants and honey bees have some of the largest odorant receptor gene families of any insect (Hansson and Stensmyr 2011; Zhou et al 2015) and massive antennal lobes with correspondingly large numbers of glomeruli (Stieb et al. 2011). Changes in olfactory investment can also be observed across short time scales. Halictid bees, for example, show repeated gains and losses of sociality (Danforth et al. 2013). A recent study of 36 species revealed that social species have a significantly higher density of antennal sensilla than secondarily solitary species (Wittwer et al. 2017). The trend for social individuals to have more sensilla was even observed within a single species that includes both social and solitary populations. It is not yet clear whether the shifts represent another example of quantitative changes in the size of preexisting OSN populations or the gain/loss of OSN types or both.

## Evolution of central olfactory circuits

The ways in which central olfactory circuits might evolve are as diverse and complex as the circuits themselves (Fig. 1), and we are not aware of any well-characterized examples. However, it is at least clear that *some* sort of central change must have occurred in the pheromone sensing system of *Heliothis* moths (see case study). Moreover, in a few other systems, central evolution is implicated by a lack of obvious peripheral change (e.g*.*, Olsson et al. 2006a,b) and/or central changes have clearly occurred but do not yet have a concrete link to behavior (e.g*.*, Auer et al. 2019). Below, we briefly present ideas on how change in central olfactory circuits may contribute to behavioral evolution, bringing in the few suggestive examples that exist. We also highlight an exciting example of central evolution in a taste circuit (Seeholzer et al. 2018) in the discussion.

### Changes in the antennal lobe

Insect antennal lobe (AL) circuits implement broad transformations that control gain and enhance the reliability and discriminability of olfactory signals before relaying them to higher brain areas (Wilson 2013). The two main types of neurons housed here may change in number, tuning, or presence/absence just like peripheral OSNs (Fig. 1). However, the determinants of tuning, and therefore, the mechanisms by which tuning may evolve are obviously quite different for central neurons. Tuning in central circuits should largely be a function of connectivity. Antennal lobe LN and PN tuning may, therefore, evolve via changes in the strength of connection to preexisting neural partners or the gain/loss of neural partners. We are not aware of any clear links between antennal lobe circuits and behavioral evolution over short time scales. However, antennal lobe circuits are likely to play a major role in moth pheromone coding. Multiglomerular PNs are a common feature of the MGC of male moths. These neurons integrate information across OSNs sensitive to different pheromones and often show selective tuning for species-specific pheromone blends (Lee et al. 2019). This makes OSN/PN connectivity in the AL a clear potential locus for the evolution of pheromone preference in moths, including in *Heliothis*.

### Changes in the lateral horn

The lateral horn plays a primary role in innate olfactory behavior (Menini 2009; Hansson 2013) and may, therefore, be a key node for between-species evolution. This is where many peripheral signals are likely integrated and ascribed innate value or meaning. Incoming PNs have stereotyped projections (Jefferis et al. 2007), and overlap of projections from distinct glomeruli provides an opportunity for localized integration by diverse third order neurons (Frechter et al. 2019; Jeanne et al. 2018, Huoviala et al. 2018). The discovery of a novel axonal branch on *D. sechellia* PNs tuned to a key noni odorant is, therefore, intriguing (Auer et al. 2019). This is just the type of change one might expect to affect innate responses. More work is needed, however, to test links to behavior and determine whether such changes are common among insects. We also note that some PNs involved in innate olfactory behavior (e.g., a subset of pheromone-sensitive PNs in moths; Lee et al. 2019) project to novel central targets, outside the lateral horn and mushroom body, that may also be sites of relevant evolutionary change.

### Changes in the mushroom body

The mushroom body is the primary center for olfactory learning and memory in insects (Menini 2009; Hansson 2013). While we have so far focused on circuit changes underlying the evolution of innate odor responses, the dynamics of learning may also evolve. After all, the ability to adjust behavior through learning may only be beneficial in certain ecological contexts, and could even be harmful. Parasitoid wasps in the genus *Nasonia* provide an interesting example. These wasps can form long-term, appetitive, olfactory associations based on the reward a female experiences when finding and laying eggs in a host fly. *N. vitripennis* will form long-term memories after a single conditioning trial, while the closely related *N. giraulti* will not (Hoedjes et al. 2012). The difference has a genetic basis (Hoedjes et al. 2014) and may have evolved in concert with host breadth (Hoedjes et al. 2012). *N. vitripennis* is a generalist that parasitizes fly species in a diverse array of habitats. Learning may help individuals hone their search image based on past egg-laying events. *N. giraulti*, in contrast, is a specialist on flies that live in bird’s nests and may thus rely more on innate responses to bird nest odors. The circuit basis of the difference in memory formation is not known, but it most likely lies somewhere in the mushroom body. Interestingly, studies of mushroom body elaboration across much longer evolutionary time scales also reveal associations with both generalist feeding ecology (Farris and Roberts 2005) and parasitoid lifestyles (Farris and Schulmeister 2011).

### Changes in neuromodulatory systems

It has long been known that a subset of neurons in the antennal lobe, mushroom body, and lateral horn are neuromodulatory, releasing neuropeptides and biogenic amines that alter olfactory processing (Hildebrand et al. 1986; Pophof 2002; Anton et al. 2007; Ignell et al. 2009; Wang et al. 2013). We would be remiss not to mention the potential role of such neurons and their partners in the evolution of olfactory behavior given the important role they play in the evolution of animal behavior more broadly (deBono and Bargmann 1998; Lim et al. 2004; Bendesky et al. 2017). As is true in other areas of the nervous system, neuromodulatory circuits within the olfactory system often function to align an insect’s behavior with its internal state. In *D. melanogaster*, for example, hungry flies are more likely to accept suboptimal food sources due to the dual action of tachykinin and short neuropeptide F (sNPF) on OSN terminals in the antennal lobe (Ko et al. 2015). sNPF increases neurotransmission from OSNs that respond to attractive food odors, while tachykinin dampens transmission from OSNs that respond to aversive odors. Although the mechanisms are less well understood, neuromodulators may also be involved in the olfactory shifts many insects experience after mating—often involving sex-specific changes in response to pheromones, oviposition host odors, and nectar host odors (e.g*.*, Saveer et al. 2012; Kromann et al. 2015). The role of neuromodulatory systems in the evolution of such state-dependent olfactory behavior is an interesting area for future work.

## Discussion

Recent years have seen a surge of interest at the intersection of evolutionary biology and neuroscience. How does evolution shape animal nervous systems? How do neural circuits evolve to help animals survive and reproduce in novel environments? Here, we review studies that address these questions across short evolutionary time scales where homologous neurons can be identified with confidence and circuit differences can often be linked to specific behaviors. Following Darwin, we imagine that the complex differences that characterize the brains of distantly related animals accumulate through processes that can also be observed in recent time (Darwin 1859).

### The relative importance of peripheral versus central evolution

The relative importance of peripheral versus central change remains a key question in the evolution of sensory systems. Does evolution tend to alter the way sensory stimuli are detected at the periphery or the way sensory information is processed centrally? If we take the literature at face value, the answer for insect olfactory systems is clearly that peripheral changes dominate—more specifically changes in the number and tuning of sensory neurons. These changes appear to reshape behavior by enhancing or diminishing neural responses to compounds with new ecological or social relevance. Nevertheless, it is currently impossible to say whether examples of central evolution are missing from the literature, because they are truly rare or because they are just more difficult to detect. Exploring central circuits is challenging even in the best model systems, let alone non-model organisms that lack neurogenetic tools. Some argue that peripheral evolution should dominate, because it is less subject to pleiotropy—it can occur with fewer negative side effects on other aspects of an organism’s biology. OSN tuning, for example, can evolve via changes in olfactory receptors and other dedicated olfactory genes that act nowhere else in the nervous system. Central circuits, in contrast, are regulated by broadly expressed genes that function throughout the nervous system. This logic mirrors a conceptually similar debate in evolutionary genetics regarding the primacy of coding versus regulatory evolution (Hoekstra and Coyne 2007; Stern and Orgogozo 2008). As is true for that debate, we suspect the ultimate answer will be nuanced and context specific. For example, the balance of peripheral and central change may depend on whether the behavior in question is coded combinatorially or through labelled lines, and whether it involves innate responses or the dynamics of learning etc. Definitive answers will rely on comprehensive dissection of olfactory evolution in tractable study systems. If and when peripheral changes are uncovered, it is critically important to keep pushing to determine whether central changes may also be important.

### How might evolutionary patterns differ in taste circuits?

Insects detect and respond to chemical cues through both their senses of smell and taste. Will patterns of taste evolution resemble those we see for olfaction? The distinct architecture of taste circuits (Vosshall and Stocker 2007) suggests there may be at least some notable differences. While olfactory circuits are optimized for combinatorial coding, taste circuits are largely organized in labelled lines for attraction and aversion. ‘Sweet’ gustatory sensory neurons (GSNs) express multiple receptors that detect sugars and other appetitive compounds, while ‘bitter’ GSNs express a diverse array of receptors that detect potentially dangerous or otherwise aversive compounds. These two types of neurons project to segregated regions of primary taste processing centers in the brain. Given this circuit architecture, one clear prediction is that evolution would occur primarily at the periphery, specifically by changing the tuning of GSNs to ensure that sweet and bitter neurons respond appropriately to newly appetitive or aversive stimuli, respectively. Indeed, some cockroaches have evolved resistance to the widespread use of glucose-baited traps via a switch in glucose sensitivity from sweet to bitter neurons (Wada-Katsumata et al. 2013). Likewise, while silkworm caterpillars are typically monophagous on mulberry leaves, loss of a single bitter receptor has led to the evolution of rare polyphagous strains that will eat apples, pears, and even soybeans (Zhang et al. 2019). Peripheral taste evolution also contributes to *D. sechellia*’s loss of contact aversion to noni acids via change in an odorant-binding protein expressed on the legs (Matsuo et al. 2007).

However, taste pathways are not only involved in the evaluation of food, but also in the detection of contact pheromones. A recent landmark study in *D. simulans* illustrated how a derived aversive response to the mating pheromone of heterospecific *D. melanogaster* females maps to a change in central, not peripheral, circuits. More specifically, there was a shift in the balance between excitation and feed-forward inhibition relayed from conserved pheromone-sensitive GSNs onto courtship decision neurons (Seeholzer et al. 2018). This fascinating example provides a general mechanism of sensory circuit evolution that may also apply to olfaction.

### The need for more direct testing of causality

Almost all the examples of olfactory circuit evolution described in this review are correlational—a change in olfactory behavior is correlated with a change in the olfactory system. In most cases the neural changes affect responses to behaviorally relevant compounds and the evolutionary distances are quite small. Both these factors make it more likely that the changes do truly contribute to novel behavior. However, manipulative experiments are still needed to rigorously establish causal relationships. The first such manipulations were recently conducted in *D. sechellia*, showing that the derived tuning of ab3A neurons is required for strong attraction to noni odor (Auer et al. 2019). The manipulation involved driving the expression of the *D. melanogaster* copy of *Or22a* in the appropriate *D. sechellia* neurons using CRISPR. Likewise, when cell-specific drivers are available for central neurons with altered tuning, it may be possible to use chemogenetic, thermogenetic or optogenetic tools to manipulate their activity and establish causality (Ding et al. 2019). However, establishing causality for more complex developmental changes in circuit architecture seems nearly impossible unless the underlying genetic mechanisms are simple and well characterized. Looking ahead, it will be exciting to see this interesting field of research mature with the CRISPR revolution enabling more rigorous causal inference in non-model systems.

## Abbreviations

OSN: Olfactory sensory neuron
GSN: Gustatory sensory neuron
PN: Projection neuron
LN: Local interneuron
OBP: Odorant-binding protein
MGC: Macroglomerular complex
