# Correction to: Evolution of olfactory circuits in insects

**DOI:** 10.1007/s00359-020-01409-7

**Published:** 2020-02-29

**Authors:** Zhilei Zhao, Carolyn S. McBride

**Affiliations:** 1 Department of Ecology and Evolutionary Biology, Princeton University, Princeton, NJ 08544 USA; 2 Princeton Neuroscience Institute, Princeton University, Princeton, NJ 08544 USA

## Correction to: Journal of Comparative Physiology A 10.1007/s00359-020-01399-6

Authors would like to correct the typo found in the fourth sentence of the introduction from “organisional” to “organismal”. The original article has been corrected.

